# FOXO Transcription Factors: Their Clinical Significance and Regulation

**DOI:** 10.1155/2014/925350

**Published:** 2014-04-03

**Authors:** Yu Wang, Yanmin Zhou, Dana T. Graves

**Affiliations:** ^1^Department of Implantology, School of Stomatology, Jilin University, Changchun 130021, China; ^2^Department of Periodontics, School of Dental Medicine, University of Pennsylvania, Philadelphia, PA 19104, USA

## Abstract

Members of the class O of forkhead box transcription factors (FOXO) have important roles in metabolism, cellular proliferation, stress resistance, and apoptosis. The activity of FOXOs is tightly regulated by posttranslational modification, including phosphorylation, acetylation, and ubiquitylation. Activation of cell survival pathways such as phosphoinositide-3-kinase/AKT/IKK or RAS/mitogen-activated protein kinase phosphorylates FOXOs at different sites which regulate FOXOs nuclear localization or degradation. FOXO transcription factors are upregulated in a number of cell types including hepatocytes, fibroblasts, osteoblasts, keratinocytes, endothelial cells, pericytes, and cardiac myocytes. They are involved in a number of pathologic and physiologic processes that include proliferation, apoptosis, autophagy, metabolism, inflammation, cytokine expression, immunity, differentiation, and resistance to oxidative stress. These processes impact a number of clinical conditions such as carcinogenesis, diabetes, diabetic complications, cardiovascular disease, host response, and wound healing. In this paper, we focus on the potential role of FOXOs in different disease models and the regulation of FOXOs by various stimuli.

## 1. Introduction

The forkhead transcription factor family is characterized by a winged-helix DNA binding motif and the forkhead domain [1]. The mammalian forkhead transcription factors of the O class (FOXOs) have four members: FOXO1, FOXO3, FOXO4, and FOXO6. FOXO1 and FOXO3 are expressed in nearly all tissues. FOXO4 is highly expressed in muscle, kidney, and colorectal tissue while FOXO6 is primarily expressed in the brain and liver [2].

Over the last decade, studies have demonstrated that FOXOs play critical roles in a wide variety of cellular processes. FOXOs transcriptionally activate or inhibit downstream target genes, thereby playing an important role in proliferation, apoptosis, autophagy, metabolism, inflammation, differentiation, and stress resistance (Table 1). Deletion of FOXOs has given insight into their function. Global deletion of FOXO1 is lethal; it causes embryonic cell death due to incomplete vascular development [3]. Global deletion of FOXO3 is not lethal but affects lymph proliferation, widespread organ inflammation [4], age-dependent infertility [3], and decline in the neural stem cell pool [5]. Global deletion of FOXO4 exacerbates colitis in response to inflammatory stimuli [6]. Global deletion of FOXO6 displays normal learning but impaired memory consolidation [7].

## 2. Regulation of FOXO Activity

**Figure 1:**
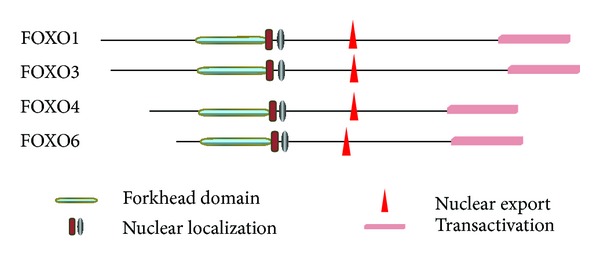
Regulatory motifs of FOXO1 (655aa), FOXO3 (673aa), FOXO4 (505aa), and FOXO6 (492aa). The functional domains are indicated: forkhead domain (teal), nuclear localization (brown/gray), nuclear export (red), and transactivation (pink).

FOXO transcriptional activity is regulated by a complex array of posttranslational modifications. These modifications can be either activating or inactivating. They alter nuclear import and export steps, modify the DNA binding affinity, and alter the pattern of transcriptional activity for specific target. FOXOs share significant sequence homology and possess four distinct functional motifs which include a forkhead, nuclear localization, nuclear export, and transactivation domains (Figure 1). These domains are highly conserved. FOXO1 and FOXO3 proteins are larger (greater than 650 amino acids) than FOXO4 and FOXO6, which are closer to 500 amino acids. FOXOs recognize two response elements: Daf-16 family member binding element (5′-GTAAA(T/C)AA) and insulin-responsive element (5′-(C/A)(A/C)AAA(C/T)AA). The core DNA sequence 5′-(A/C)AA(C/T)A-3′ is recognized by all FOX-family members. Although FOXOs recognize both Daf-16 family member binding element and the insulin response element, they have a higher affinity for the former [8]. The transport of FOXO proteins through the nuclear pore is dependent on active-transport mechanisms. The presence of a nuclear localization sequence is a prerequisite for maintaining proteins in the nucleus, whereas a nuclear export sequence maintains proteins in the cytosol. FOXO proteins have both a nuclear localization sequence and a nuclear export sequence within the C-terminal DNA binding domain. Kinases and interactions with other proteins modulate the effectiveness of these nuclear localization sequences and nuclear export sequences, which forms the basis of FOXO shuttling in and out of the nuclear compartment. The cytoplasmic sequestration of FOXO proteins is mediated by a combination of binding partners and changes in the properties of FOXO. The chaperone protein 14-3-3 binds to FOXO factors in the nucleus and allows their active export [9]. It also blocks the nuclear localization signal to prevent FOXO re-entry into the nucleus [10].

### 2.1. Phosphorylation

FOXOs are targeted for phosphorylation by several protein kinases, which modify different sites on FOXOs that alter their subcellular location, DNA binding affinity, and transcriptional activity [36, 37]. The PI3K pathway is an important regulator of FOXO activity. The serine-threonine kinases, protein kinase B (AKT), and serum/glucocorticoid inducible kinase (SGK) are important downstream components of insulin/PI3 kinase pathway [38]. AKT/SGK protein kinases phosphorylate FOXO1 and FOXO3 at well-defined sites, which increase the association with 14-3-3 proteins. This in turn results in the translocation of FOXO proteins from the nucleus to cytoplasm leading to their transcriptional inactivation [39]. Growth factor-activated protein kinases such as casein kinase 1 also phosphorylate and potentiate FOXO1 export to the cytoplasm by directly increasing the interaction between FOXO and the export machinery and Ran and Exportin/Crm1 [40, 41]. These also include extracellular signal-regulated kinase, IkB kinase beta, and cyclin-dependent kinase 2. Other protein kinases function to promote nuclear localization and increase FOXOs transcriptional activity. These include JNK, p38, AMPK, cyclin-dependent kinase 1, and macrophage stimulating 1. The increased nuclear localization is accomplished in part by disrupting FOXO binding to 14-3-3 proteins [37]. FOXO6 is primarily localized to the nucleus as it lacks the C-terminal AKT phosphorylation site. Phosphorylation of FOXO by AKT also disrupts FOXO interactions with DNA. The phosphorylation of FOXO at the second of the three AKT/SGK sites (S256 for FOXO1) introduces a negative charge in the positively charged DNA-binding domain, thereby inhibiting DNA binding. FOXO1 is also regulated through the insulin signaling substrates 1 and 2 of the insulin signaling cascade [38]. During insulin stimulation, FOXO1 is phosphorylated by AKT and accumulates in the cytosol [38].

### 2.2. Acetylation

Similar to phosphorylation, acetylation has been shown to both promote and decrease FOXO transcriptional activity and to mediate different biological functions of FOXOs [36, 37]. The effect of acetylation on FOXOs is controlled by the histone acetyltransferase and histone deacetylases. Several lysines are acetylated in FOXOs. FOXO3 is acetylated at K242, K259, K271, K290, and K569 in the presence of stress stimuli. Acetylation at K222, K245, K248, K262, K265, K274, and K294 of FOXO1 was also reported to regulate its DNA binding affinity and sensitivity to AKT phosphorylation. Acetylation at K242, K245, and K262 of FOXO1 is sufficient to attenuate its transcriptional activity. Deacetylation at K186, K189, and K408 by histone deacetylases plays an important role in regulating FOXO4 transcriptional activity. It is reported that more highly acetylated forms of FOXO3 favor expression of proapoptotic genes, (Bim, TRAIL, and FasL), while the more deacetylated forms favor expression of antioxidant and cytoprotective genes. FOXO DNA binding activity is reduced by acetylation and enhanced by deactylation [36, 37, 42, 43]. The binding of CREB binding protein (CBP) and its paralog p300 to FOXOs is essential for transactivation of target genes. However, the acetylation itself attenuates FOXO transcriptional activity [36, 37]. In FOXO1, CBP induced acetylation at two basic residues, Lys242 and Lys245 located in the C-terminal region of the DNA binding domain, has been shown to reduce its DNA binding affinity and transcriptional activity [44]. Moreover reactive oxygen species stimulate formation of cysteine-thiol disulfide-dependent complexes between FOXO4 and p300/CBP acetyltransferase, which reduces FOXO4 induced cell cycle arrest and enhances FOXO4 induced apoptosis [45]. Silent information regulator 2 belongs to the sirtuin family of NAD-dependent deacetylases, which respond to metabolic changes in the cellular environment, including the availability of nutrients/energy, and stress stimuli [46]. Sirt1 binds to FOXOs and catalyze its deacetylation in an NAD-dependent manner and thereby increase its transactivation activity by regulating its DNA binding at specific target genes [42].

### 2.3. Ubiquitination

Ubiquitination also plays a dual role in the regulation of FOXO proteins. FOXO, like many other proteins, is targeted for proteasome degradation through polyubiquitination. Several ubiquitin E3 ligases are necessary for the ubiquitination of FOXOs [39], which leads to FOXO1 degradation. In contrast proteasome inhibitors can block this degradation and increase FOXO expression [47]. Elevated SKP2 (an oncogenic subunit of the Skp1/Cul1/F-box protein ubiquitin complex) levels are found in a wide variety of human cancers, which can recognize the Ser256 phosphorylated FOXO1 and degrade it by polyubiquitination [48]. In addition to this canonical role for ubiquitination in protein degradation, monoubiquitination also plays a role in FOXO regulation. Monoubiquitination of FOXOs has the opposite effect and increases FOXO nuclear localization and induced transcription activity [49]. For example, oxidative stress stimulates relocalization of FOXO4 into the nucleus and the subsequent activation of FOXO-dependent transcription by inducing the monoubiquitination of FOXO4 at K199 and K211 [43, 50, 51]. Another mechanism is a ROS induced formation of a complex of FOXO4 and the nuclear import receptor transportin-1 that facilitates nuclear localization [52].

### 2.4. Interaction of FOXOs with Protein Partners

FOXOs can associate with a variety of protein partners, activating or repressing diverse target genes. The transcription factors and the co-activators expressed in a particular cell type are thus critical in determining the functional FOXO activity. FOXOs themselves may regulate expression of target genes without directly binding to DNA. For example it has been shown by overexpression that a FOXO mutant which lacks DNA binding activity was still able to regulate target gene expression. This result suggests that FOXO may regulate a subset of target genes through interaction with other transcription factors. It is also possible that this result reflects FOXO1 activity only when pharmacologic levels of FOXOs are present by overexpression [53]. In some cases FOXOs bind to other factors to regulate downstream transcription activity. For example, FOXO3 and Runx3 interact and bind concomitantly to the promoter of Bim to promote apoptosis. The interaction of Runx3 and FOXO3 is indispensable for Bim expression and apoptosis in mouse embryonic fibroblasts and gastric cancer cells [54]. In another example FOXO proteins have been shown to interact with *β*-catenin. It has been suggested that when FOXO1 binds to *β*-catenin, *β*-catenin is not available to bind to T cell factor, thus reducing T cell factor activity [55]. Thus, FOXO1 competes with T cell factor for *β*-catenin and negatively impacts the capacity of *β*-catenin to stimulate bone formation.

### 2.5. Regulation of FOXO1 by TNF-*α*

Tumor necrosis factor-alpha (TNF-*α*) is a potent proinflammatory and proapoptotic mediator that plays an important role in several normal and disease processes by activating several different signaling pathways. FOXO1 is strongly activated by TNF-*α* both in vitro and in vivo [12]. In a chronic low-grade inflammatory environment, FOXO1 activates the C/EBP*β* gene transcription through directly binding to its promoter in adipocytes, thereby increasing the proinflammatory genes expression such as MCP-1 and IL-6 [26]. This binding is inhibited after insulin stimulation. However, the recruitment of FOXO1 onto the C/EBP*β* gene promoter in the presence of insulin is partially restored by pretreatment with TNF-*α* [26]. TNF-*α* also enhances FOXO1 activity by reducing an inhibitory signal. TNF-*α* inhibits AKT-mediated phosphorylation of FOXO1 in adipocytes by reducing phosphorylation of insulin receptor substrate-1 on tyrosine residues thereby diminishing the negative effect of insulin receptor signaling [26].

### 2.6. Upstream Regulation of FOXO1 by LPS

Lipopolysaccharide (LPS) is a proinflammatory bacterial virulence factor found in the cell wall of Gram-negative bacteria. LPS stimulates FOXO expression, nuclear localization, and FOXO-mediated gene transcription. LPS induced inflammatory cytokine expression is mediated, in part through FOXO transcription factors [27]. LPS treatment impairs the ability of insulin to phosphorylate FOXO1 in cultured macrophages. FOXO1 activity may explain the abnormal production of proinflammatory cytokine IL-1*β* and in conditions where there is insulin resistance [27]. FOXO1 promotes inflammation by enhancing Tlr4-mediated signaling in mature macrophages in response to LPS. However, LPS signaling induces Akt, which leads to rapid phosphorylation and nuclear export of FOXO1. While FOXO1 increases Tlr4-mediated inflammatory signaling, the Tlr4-PI3K-AKT pathway in turn inactivates FOXO1 transactivation and limits the inflammatory response. Insulin signaling increases AKT activity to further reduce FOXO1 activation. This negative feedback represents a self-limiting mechanism that contributes to the overactivation of the innate immune response [56]. Thus, in cells where there is insulin resistance, this inhibitory component is reduced. FOXO1 overstimulation of inflammation is also modulated by a feedback mechanism involving the mTOR pathway [56]. Rictor, a key component of mTORC2, plays a role in controlling the inflammatory response by reducing FOXO1 activation by LPS. These findings suggest that mTORC2 activates a negative feedback loop after LPS stimulation to suppress FOXO1, which limits inflammatory cytokine expression [56].

### 2.7. Cooperative Regulation of Inflammatory Genes by NF-*κ*B and FOXO1

FOXO1 plays a cooperative role in inflammatory signaling through NF-*κ*B. This cooperation couples proinflammatory cytokine production with insulin resistance and is thought to contribute to greater inflammatory signaling in obesity and type 2 diabetes [27]. Macrophage production of IL-1*β* is governed by NF-*κ*B [57, 58]. NF-*κ*B in the active state consists of a dimer and in the inactive state a trimer that contains the active dimer plus an inhibitor I*κ*B subunit. In the presence of inflammatory stimuli, I*κ*B is phosphorylated and dissociates from the active NF-*κ*B dimer, which then translocates to the nucleus to promote inflammation [58]. Several genes such as the IL-1*β* promoter contain both FOXO1 and NF-*κ*B response elements. FOXO1 enhances IL-1*β* expression when the NF-*κ*B dimer simultaneously binds to its response elements. Both FOXO1 and NF-*κ*B are needed to induce IL-1*β* transcription. When FOXO1 is inhibited by insulin signaling, expression of IL-1*β* is reduced. When insulin signaling is reduced, the level of inflammation increases because of greater FOXO1 binding to the promoter sites of inflammatory genes. Thus, FOXO1 acts to amplify NF-*κ*B induced inflammation and inflammation is reduced if this amplifying component is inhibited [59, 60]. For the chemotactic ligand CCL20, it is found that FOXO1 overexpression increases binding of the active NF-*κ*B dimer, while FOXO1 silencing decreases NF-*κ*B binding to its response element. Since FOXO1 does not bind CCL20 promoter directly, it is proposed that FOXO1 may serve as a coactivator of NF-*κ*B in the nucleus to amplify NF-*κ*B signaling [28]. Thus, in some cases such as IL-1*β*, FOXO1 binds to a response element nearby the NF-*κ*B binding element to enhance transcription, whereas, in CCl20, FOXO1 is thought to physically interact with NF-*κ*B and enhance NF-*κ*B induced CCL20 transcription. In cases where there is deficient inhibition of FOXO1 through insulin resistance, inflammation may be enhanced by greater cooperation between NF-*κ*B and FOXO1. However, in a colonic injury and inflammation model, it was also found both in vivo and in vitro that FOXO4 inhibits the transcriptional activity of NF-*κ*B by reducing its DNA binding activity [6].

## 3. FOXO1/FOXOs and Their Clinical Significance

**Figure 2:**
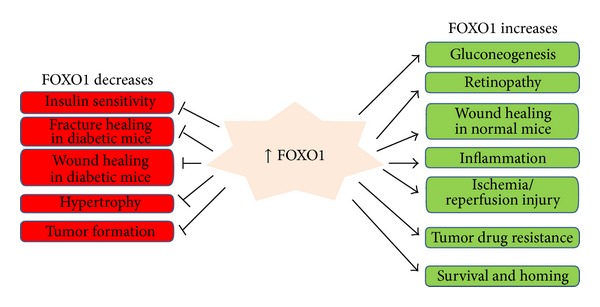
Explanation of FOXO1 clinical significance. FOXO1 is the best-studied member of FOXO subfamily. FOXO1 function has been investigated in the tissues and cells of various genetically modified mice of different disease models such as diabetic complications, cardiomyopathy, and carcinogenesis. FOXO1 has been shown to enhance or diminish the clinical events either based on animal studies or projection from in vitro studies.

FOXO1 is the best-studied member of FOXO subfamily. Loss and gain of FOXO1 function have been investigated in the tissues and cells of various genetically modified mice of different disease models (Figure 2).

### 3.1. Gluconeogenesis

Under fasting conditions, the liver provides energy by releasing glucose into the bloodstream. Gluconeogenesis is regulated over the long term primarily through alterations in the expression of three major gluconeogenic enzymes: G6Pase, fructose-1,6-biphosphatase, and PEPCK [61]. PEPCK is the rate-limiting enzyme that phosphorylates oxaloacetate to form phosphoenolpyruvate, whereas G6Pase promotes the dephosphorylation of glucose-6-phosphate, allowing for the release of newly synthesized glucose into the bloodstream. Chronic expression of an active FOXO1 mutant in the liver of transgenic mice leads to increased expression of genes described above that are involved in gluconeogenesis, resulting in elevated plasma glucose levels. A major regulator of G6Pase and PEPCK, and consequently gluconeogenesis, is PGC1*α* [62]. FOXO1 interacts with PGC1*α*, thereby increasing the expression of G6Pase and PEPCK. Transgenic mice that overexpress FOXO1 have an impaired ability to regulate blood glucose levels [63]. Liver-specific inactivation of the FOXO1 gene reduces glucose levels because of decreased hepatic glucose production [64]. Dominant-negative inhibition of FOXO1 in mice also decreases fasting blood glucose levels by suppressing the expression of the gluconeogenic genes G6Pase and PEPCK [65]. In the fed state, transcription of FOXO1 dependent genes is antagonized by insulin induced AKT phosphorylation [66].

### 3.2. Insulin Sensitivity and Lipid Metabolism

FOXO1 functions as a negative transcriptional modulator of insulin sensing genes, which reduces insulin sensitivity. Conditional deletion of FOXO1 in insulin-resistant mutant mice restores insulin sensitivity and rescues the diabetic phenotype by reducing hepatic expression of gluconeogenic genes (e.g., G6pc and Pck1) and by increasing adipocyte expression of insulin-sensitizing genes (e.g., PPAR*γ*, Lep, and Slc2a4) [67]. The transcription factor pancreatic and duodenal homeobox 1 (Pdx1) plays a crucial role in *β*-cell growth and function. It is regulated by another forkhead transcription factor, FOXA2. FOXO1 and FOXA2 share common DNA binding sites in the Pdx1 promoter. FOXO1 competes with FOXA2 for binding to Pdx1 promoter, resulting in inhibition of Pdx1 transcription [68]. Overexpression of constitutively active FOXO1 targeted to the liver and pancreatic *β*-cells results in diabetes arising from a combination of increased hepatic glucose production and inhibited *β*-cells compensatory growth due to decreased Pdx1 expression [67]. FOXO1 in osteoblasts has also been linked to regulation of serum glucose levels. It has been proposed that deletion of FOXO1 in osteoblasts increases insulin sensitivity and insulin production through increased osteocalcin expression and decreased expression of Esp [69]. Patients with diabetes suffer disproportionately from impaired lipid metabolism and FOXO1 controls aspects of lipid metabolism in the diabetic liver. FOXO1 ablation in the liver increases serum levels of very low density lipoproteins, cholesterol, and plasma free fatty acids, three hallmarks of the diabetic condition [70]. These findings suggest that FOXO1 can protect against excessive hepatic lipid production during hyperglycemia and may indicate that insulin treatment, which inhibits FOXO1, may indirectly worsen lipid abnormalities in diabetics [70].

### 3.3. Diabetic Complications

Patients with diabetes have an increased risk of developing a number of serious health problems such as retinopathy and impaired fracture. It has been proposed that diabetes-enhanced activation of FOXO1 is associated with several diabetic complications. FOXO1 promotes diabetic retinopathy by increasing apoptosis in microvascular endothelial cells and pericytes [29]. In vivo experiments indicate that diabetes increases FOXO1 mRNA levels, DNA binding activity, and nuclear translocation mediated by TNF-*α* in retinal microvascular cells. Knockdown of FOXO1 by siRNA in vivo diminishes the loss of retinal microvascular endothelial cells and pericytes, the first step in diabetic retinopathy [29]. In vitro mRNA profiling suggests that FOXO1 mediates high-glucose induced mRNA expression of genes that modulate endothelial cell activation such as CCL2 and CCL5, enhances apoptosis by increasing mRNA levels of BCL2 and CASP3, and increases the basal expression of genes that affect angiogenesis such as ITGA5 and ITGAV-M [29]. In vitro TNF-*α* and an advanced glycation end-product, which are elevated in diabetic retinopathy, induce pericyte apoptosis through activation of the transcription factor FOXO1 [13].

FOXO1 has been linked to impaired diabetic fracture healing. In vivo experiments demonstrate that diabetes enhances FOXO1 DNA binding activity and increases FOXO1 nuclear translocation in chondrocytes [14]. Studies suggest that this in turn causes expression of inflammatory and resorptive factors leading to greater loss of cartilage in diabetic fractures [14]. In vitro FOXO1 mediates TNF-*α* induced expression of proosteoclastogenic factors in chondrocytes (TNF-*α*, RANKL, M-CSF, IL-1*α*, and IL-6) and the chemokine CCL4, which is linked to a burst of osteoclast activity and accelerated loss of cartilage in diabetic fractures [14, 71]. FOXO1 also promotes TNF-*α* induced apoptosis and upregulates proapoptotic genes in chondrogenic cells including caspase-3, caspase-8, caspase-9, and TRAIL [72].

### 3.4. Wound Healing

**Figure 3:**
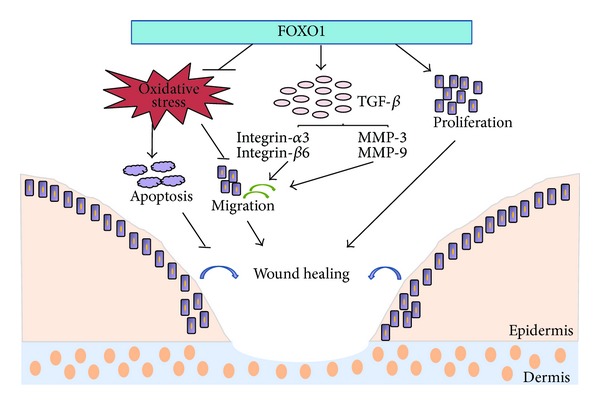
Mechanisms of FOXO1 in normal wound healing. The normal wound healing process is initiated by the integration of multiple intercellular signals (cytokines and chemokines) released by keratinocytes and other cells. FOXO1 is required for keratinocyte transition to a wound-healing phenotype. FOXO1 in vivo is needed for keratinocyte expression of transforming growth factor-*β*1 (TGF-*β*1) expression, induction of TGF*β*1 downstream targets (integrin-*α*3 and -*β*6 and MMP-3 and -9), and migration. Migration (bold arrow) is particularly important in wound healing. FOXO1 is also needed to protect keratinocytes from oxidative stress, which contributes to keratinocyte migration and survival during normal wound healing. This is adapted from [19].

FOXO1 plays a positive role in wound healing in normal mice [19] (Figure 3). It coordinates the response of keratinocytes to wound healing through upregulation of TGF-*β*1 and its downstream targets, integrin-*α*3 and -*β*6, and MMP-3 and -9 which are needed for keratinocyte migration. FOXO1 also functions in keratinocytes to reduce oxidative stress that is necessary to maintain cell migration and prevent cell death in a TGF*β*1 independent manner. However, in the diabetic wounds, FOXO1 has been linked to impaired wound healing. In diabetic wounds FOXO1 DNA binding activity and nuclear translocation are driven by TNF-*α* and associated with higher levels of apoptosis and reduced proliferation of fibroblasts [73, 74]. In vitro experiments suggest that FOXO1 may negatively affect fibroblasts through expression of proapoptotic factors [12].

### 3.5. Cardiomyopathy

Autophagic vacuoles are found in cardiomyocytes in ischemic [75] and in cardiomyopathic failing hearts [76]. Autophagy may also mediate the regression of cardiac hypertrophy [77]. It is an evolutionarily conserved process for the degradation of cytoplasmic components. Autophagy may play a protective role under some circumstances but also may have a causative role in cell death. Several reports confirm that FOXO1 plays both a positive and negative role in autophagy related cardiomyopathy. In vivo experiments establish the fact that cellular stress such as ischemia/reperfusion induces autophagy in the heart with concomitant increased nuclear localization and FOXO activity [78]. FOXO1 can induce autophagy and reduce cardiomyocyte cell size in vitro by binding to promoter sequences of autophagy pathway genes Gabarapl1 and Atg12 and induce their expression [24]. FOXO1 also mediates regression of cardiac hypertrophy by affecting autophagy [79]. FOXOs reduce cardiac hypertrophy by inhibiting the calcineurin/nuclear factor of activated T cells pathway, which is a key signaling cascade that promotes cardiac hypertrophy [25].

### 3.6. Carcinogenesis

FOXO1 acts as a tumor suppressor. Inactivation of FOXO1 has been documented in many types of human cancer. FOXO1 activation inhibits tumor cell survival by inducing apoptosis in prostate cancer and glioma cells through upregulating proapoptotic factors [80, 81]. Prostate cancer patients with regional lymph node involvement often experience disease progression to other organs, with the bone as the predominant site [82]. Increased FOXO1 activation may limit the metastasis of the prostate cancer cells to other organs by inhibiting the migration and invasion through inhibition of Runt-domain containing protein Runx2 transcriptional activity [83]. Runx2 is normally expressed in mesenchymal cells committed to the lineage of osteoblasts. However, it should be noted that under some conditions FOXO1 can induce the expression of genes that impart resistance to chemotherapy [84].

Compared to a single deletion, deletion of multiple FOXOs creates a more severe susceptibility to thymic lymphomas and hemangiomas. In vivo experiments demonstrate that the lymphomas display an enrichment of the null alleles for three FOXO genes accompanied by a marked decrease of FOXO expression. There is reduced formation of lymphomas in genotypes that retain at least one active FOXO allele. In addition, complete loss of FOXO gene function in thymocytes predisposes to lymphomagenesis through mechanisms that enhance cellular proliferation and survival [85].

### 3.7. Oxidative Stress

FOXO1 plays an important role in protection of cells against oxidative stress. Under normal conditions FOXO1 induces expression of antioxidant genes to decrease apoptosis [20]. This function of FOXO transcription factors is important in long term survival of hematopoietic stem cells as shown by increased hematopoietic stem cell apoptosis with deletion of FOXO1, FOXO3, and FOXO4 [86]. Diabetes is caused by pancreatic *β*-cell failure. FOXO1 can protect *β*-cells against oxidative stress in the pancreas [87]. Oxidative stress is also important in wound healing. We have shown that in normal wound healing FOXO1 functions to reduce oxidative stress in keratinocytes that is necessary to maintain cell migration and prevent cell death [19]. In contrast, FOXO1 appears to promote cell death when oxidative stress is more extreme such as in tissues that are affected by diabetic complications [88]. In the latter, FOXO1 may have a destructive rather than a protective role [88].

### 3.8. Innate Immune Response

FOXO1 has been shown to enhance inflammation. FOXO1 promotes inflammation by increasing expression of several proinflammatory genes. FOXO1 mediates expression of proinflammatory cytokines in response to high glucose, TNF, and LPS stimulation [88]. Macrophages from insulin-resistant obese db/db mice have increased FOXO1 activation, which is associated with elevated production of IL-1*β*. Moreover, FOXO1 promotes IL-1*β* expression by binding to the IL-1*β* promoter [27]. FOXO1 also increases Tlr4 expression [28]. Since FOXO1 is inhibited by insulin, a reduction in insulin signaling will tend to enhance FOXO1 activation and to promote inflammation. This highlights the role of FOXO1 as a key molecular proinflammatory transcription factor in the context of obesity and insulin resistance. In dendritic cells and embryo fibroblasts FOXO1 mediates LPS stimulated IL-6 and IL-12 expression but reduces IL-10 production [30]. We have recently found that FOXO1 expression by dendritic cells is needed for dendritic cell homing to lymph nodes and optimal induction of an adaptive immune response to bacterial challenge (Dong G. et al., unpublished data).

### 3.9. Adaptive Immunity

Naive T lymphocytes travel between the bloodstream and secondary lymphoid organs. Several molecules are required for this constitutive trafficking. FOXO1 increases expression of receptors that control T cell trafficking to secondary lymphoid organs tissues and include L-selectin, EDG1, and EDG6, the chemokine receptor CCR7, and the transcription factor Klf2 [89, 90]. Survival and homeostasis of T cells are influenced by the IL-7. FOXO1 controls T cell tolerance and naive T cell homeostasis through the induction of IL-7R expression. It binds to the promoter of IL7r gene and may promote expression by interacting with other nuclear factors (e.g., GABP and Gfi-1) [91]. FOXO1 also regulates the homing of peripheral B cells through upregulation of L-selectin and regulates class-switch recombination in peripheral B cells [92]. FOXO1 plays a role in T cells by enhancing survival of CD8 memory T cells [93].

Regulatory T cells (Tregs) play an indispensable role in maintaining immunological unresponsiveness to self-antigens and in suppressing excessive immune responses deleterious to the host. Tregs are produced in the thymus. Formation of Treg requires FOXO transcription factors that regulate expression of the transcription factor FOXp3 [94]. FOXO1-deficient T cells stimulated in the presence of TGF-*β* are misdirected to a Th1 cell phenotype, demonstrating that FOXO1 is necessary for TGF-*β* induced differentiation of Treg cells [94]. Moreover, these studies suggest that the absence of FOXO1 through loss of Treg cells increases the likelihood of autoimmunity.

### 3.10. Osteoblasts

Recent evidence suggests that FOXO factors play a fundamental role in skeletal homeostasis by upregulating antioxidant enzymes [11, 20]. Deletion of FOXO1 in osteoblasts results in decreased expression of antioxidants such as glutathione. Consistent with this, conditional deletion of FOXO factors (FOXO1/3/4) in bone results in increased oxidative stress, loss of osteoblasts, and decreased bone mass indicating that FOXO factors are indispensable for skeletal homeostasis because of their stimulation of antioxidant defense mechanisms [11, 20]. Moreover, the deletion of multiple FOXOs has greater impact than deletion of individual FOXOs. It has also been reported that FOXO1 deletion inhibits formation of a mineralized matrix in vitro by osteoblastic cells and that FOXO1 interacts directly with the Runx2 promoter [95]. Moreover, FOXO1 overexpression increases the expression of osteogenic markers such as Runx2, alkaline phosphatase, and osteocalcin indicative of enhanced bone formation [96]. FOXO1 also promotes protein synthesis in osteoblasts through direct regulation of ATF4, a transcription factor required of amino acid import and protein synthesis [11]. These results indicate that FOXO1 plays an important role in promoting the differentiation or activity osteoblasts, which is critical for bone formation.

In the previous examples, FOXO1 plays a positive role in bone formation by enhancing differentiation or activity of osteoblasts and protecting these cells through induction of antioxidants. However, under other conditions, FOXO1 may have a negative effect on bone by affecting Wnt signalling. FOXOs can attenuate Wnt/*β*-catenin signaling by diverting *β*-catenin from the nucleus [97]. In vivo deletion of FOXOs in progenitors of osteoblasts and adipocytes increases osteoblast numbers and bone mass. This is thought to occur by increasing proliferation of osteoprogenitor cells and enhancing bone formation by reducing FOXO1 interference of Wnt/*β*-catenin signaling [97]. In addition, FOXO1 may contribute to immune-mediated inhibition of bone formation by promoting apoptosis of osteoblasts [98]. Thus, the effects of FOXO transcription factors on bone are complex and may depend upon specific conditions.

## 4. FOXO3 and Its Clinical Significance

### 4.1. Cardiovascular Disease

Vascular smooth muscle cell proliferation and migration contribute significantly to atherosclerosis, postangioplasty restenosis, and transplant vasculopathy. FOXO3 is thought to play a positive role in limiting these diseases by inhibiting smooth muscle cell proliferation and activation. In vivo overexpression of FOXO3 increases p27 (kip1) in vascular smooth muscle cells and inhibits neointimal hyperplasia [99]. Cysteine-rich angiogenic protein 61 is an immediate early gene expressed in these cells upon growth factor stimulation. Angiogenic protein 61 expression is associated with postangioplasty restenosis. Both in vivo and in vitro experiments confirmed that FOXO3 inhibits vascular smooth muscle cells proliferation and neointimal hyperplasia by inhibiting the expression of cysteine-rich angiogenic protein 61 through a FOXO binding motif in the cysteine-rich angiogenic protein 61 promoter region [100]. Similar to FOXO1, FOXO3 has been postulated to play both a positive and negative role in autophagy related cardiomyopathy such as ischemic and cardiac hypertrophy. In vivo overexpression of FOXO3 reduces the cardiomyocyte size by increasing autophagosome formation, expression of atrogin-1, and autophagy-related genes (LC3, Gabarapl1, and Atg12) [78, 101].

### 4.2. Carcinogenesis

In carcinogenesis, FOXO3 and FOXO1 both suppress tumor growth. Restoring the activity of FOXO3 promotes tumor cell death. For example, the anticancer drugs such as STI571 and paclitaxel inhibit tumor growth by increasing levels of Bim expression through upregulation of FOXO3 in chronic leukemia cells and breast cancer cells [102, 103]. Another mechanism involves FOXO3 downregulation of Myc. Since Myc enhances tumor cell proliferation and survival, its downregulation by FOXO3 is antitumorigenic [104]. In some cases FOXO3 has the opposite effect of enhancing survival of drug-resistant tumor cells through its antioxidant effect like that of FOXO1 in this process [105].

### 4.3. Oxidative Stress

Similar to FOXO1, FOXO3 also plays an important role in protection of cells against oxidative stress. FOXO3 increases the levels of manganese superoxide dismutase (MnSOD) in mitochondria, which removes superoxide radicals. In vivo experiments suggestthat FOXO3 protects against oxidative stress by increasing MnSOD expression and production of catalase and peroxiredoxin III [21]. FOXO3 also protects erythropoiesis against oxidative stress [106] and decreases oxidative stress in cardiac fibroblasts [22]. FOXO3 is the predominant FOXO isoform expressed in neural stem/progenitor cells. Among the FOXO3-regulated genes in neural stem/progenitor cells are antioxidants [107]. In vitro experiments suggest that FOXO3 deletion in these cells impairs two major metabolic modules (glycolysis and Gln metabolism), which contribute to oxidative stress. FOXO3 is also critical for hematopoietic self-renewal. In vivo experiments establish that FOXO3 deletion in hematopoietic stem cells increases ROS and impairs their hematopoietic capacity [108].

### 4.4. Adaptive Immunity

FOXO3 inhibits T cell proliferation and induces T cell apoptosis. FOXO3 induces T cells apoptosis through upregulation of Puma and Bim after IL-12 withdrawal [109]. FOXO3 also suppresses T cell proliferation and T cell activation, which has been shown to prevent autoimmunity. In vivo deletion of FOXO3 leads to spontaneous lymph proliferation associated with inflammation, which correlates with the presence of hyperactivated helper T cells along with more production of Th1 and Th2 cytokines [4]. FOXO3 also restrains the magnitude of T cell in immune responses by inhibiting the capacity of dendritic cells to produce IL-6 [110]. Similar to T cells, FOXO3 induces B cell apoptosis through upregulation of both proapoptotic genes such as Bim and antiproliferative genes such as Rb2 [111]. FOXO3 also regulates FOXp3 expression that is needed to generate Treg cells [112]. FOXO3 deficiency results in defective TGF-*β*-driven FOXp3 induction. Thus, FOXO3 promotes transcription of the FOXp3 gene in Treg cells similar to that of FOXO1. In addition, the absence of FOXO3 exacerbates the loss of Treg cell formation in mice with FOXO1 deletion [94] so that the impact when both are absent is greater than the loss of FOXO1 alone.

### 4.5. Aging and Reproduction

*C. elegans* is the most extensively studied organism in aging research and DAF-16, a homolog of mammalian FOXO genes, affects longevity by extending* C. elegans* lifespan [31]. In mammalian cells deletion of FOXO1 or FOXO3 limits expression of antioxidants to enhance oxidative stress and cell injury associated with aging. Extension of cellular lifespan that depends upon the prevention of cell senescence also may require the negative regulation of AKT to allow for the activation of FOXO3 [32].

In recent years, a number of in vivo mouse genetic experiments prove that FOXO3 functions in suppressing the initiation of follicular growth and control reproductive potential. FOXO3 deletion results in early depletion of the primordial follicle pool so that young female mice have normal size litters but reach menopause more quickly. Constitutively active FOXO3 expressed in transgenic mice suppresses follicular maturation and largely prevents ovulation [34]. DAF-16/FOXO may also affect the delivery of polyunsaturated fatty acids to oocytes which may influence ovulation and reproduction [35].

## 5. FOXO4 and Its Clinical Significance 

### 5.1. Diabetic Complications

Diabetic nephropathy is the leading cause of renal failure. It is thought that hyperglycemia activates multiple downstream signaling pathways in the diabetic kidney, which contributes to the development of diabetic nephropathy [113]. Advanced glycation end-products contribute to the development of diabetic nephropathy [114]. FOXO4 mediates podocyte apoptosis induced by advanced glycation end-products by increasing the expression of the proapoptotic gene Bim [115].

### 5.2. Cardiovascular Disease

During atherosclerosis, vascular smooth muscle cells migrate from the medial layer of the blood vessel wall to the intimal layer, which requires of the action of matrix metalloproteinases (MMPs) [116]. MMP9 is required for smooth muscle cell migration during the development of restenotic and atherosclerotic lesions. FOXO4 activates transcription of the MMP9 gene in response to TNF-*α* signaling. Inhibition of FOXO4 expression reduces the ability of vascular smooth muscle cells to migrate in vitro and in vivo deletion of FOXO4 inhibits neointimal migration of these cells associated with reduced MMP9 expression [117]. These studies suggest that FOXO4 is as a potential therapeutic target for combating proliferative arterial diseases [117].

### 5.3. Carcinogenesis

FOXO4 functions as a tumor suppressor in the development and progression of cancer. The expression of FOXO4 is significantly decreased or deleted in colorectal cancer tissue [118]. Strong expression of HER2, a receptor tyrosine kinase oncogene in cancers, has been associated with a poor prognosis. Constitutively active FOXO4 can reduce tumor onset, size, and progression in nude mice transplanted with Her2-positive breast cancer cells by inhibiting AKT activity, regulating P27 kip1 stability and suppressing HER2-mediated tumorigenicity [119]. FOXO4 can also inhibit growth factor-mediated tumor metastasis in cholangiocarcinoma by enhancing ANXA8 expression, which inhibits the migratory and metastatic characteristics of cholangiocarcinoma cells [120].

## 6. FOXO6 and Its Clinical Significance

### 6.1. Neurons

FOXO6 can promote the development and function of the adult central nervous system. Neuronal polarity is essential for normal brain development. FOXO6 plays a critical role in axodendritic polarization of undifferentiated neurites in a switch from unpolarized to polarized neuronal morphology by increasing expression of the protein kinase, Pak1 [121]. Memory consolidation is a process that stabilizes memory after initial acquisition. FOXO6 promotes memory consolidation in vivo by regulating neuronal connectivity in the hippocampus. During learning FOXO6 induces the expressions of genes that orchestrate proper synaptic number and function leading to correct neuronal connectivity in the hippocampus [7].

### 6.2. Gluconeogenesis

Similar to FOXO1, FOXO6 can promote gluconeogenesis, which is inhibited by insulin signaling. Elevated FOXO6 activity in the liver augments gluconeogenesis and raises fasting blood glucose levels through increased G6pase expression. In contrast, hepatic FOXO6 depletion suppresses gluconeogenesis, resulting in fasting hypoglycemia. Insulin inhibits FOXO6 activity by inducing its phosphorylation and blocking its transcriptional activity. FOXO6 becomes deregulated in the insulin-resistant liver, accounting for its enhanced activity in promoting gluconeogenesis and correlating with the pathogenesis of fasting hyperglycemia in diabetes [122].

## 7. Conclusion

FOXO transcription proteins modulate several cellular functions including proliferation, apoptosis, stress resistance, inflammation migration, and metabolism through regulation of multiple transcriptional targets. FOXO function has been investigated in various disease models. In some diseases such as diabetes or diabetic complications wound benefits from inhibiting FOXOs since deletion or knockdown of FOXOs has a positive effect. However, FOXOs also have protective roles and can play an important role in differentiation, which may be useful in promoting health. For example, FOXO agonists could potentially be used to protect and prevent loss of stem cells that diminish with aging. FOXOs promote the differentiation of Treg cells that play a critical role in preventing autoimmune disease. FOXOs also enhance normal wound healing, protect against cardiovascular disease, protect against oxidative stress associated with aging, protect against ovarian follicle depletion, and have antitumor activities. FOXOs agonist in these situations may be beneficial. In light of the different cellular functions, regulation of FOXO transcription factors by antagonists in some disease states or agonists in normal and disease conditions may be useful in treating or preventing a wide variety of disorders. A further understanding of their function will provide essential insight into both basic and clinical processes.

## Figures and Tables

**Table 1:** Cellular functions regulated by FOXO transcription factors.

FOXO	Cellular function	Pathway or target	Reference
FOXO1, FOXO3, and FOXO4	Proliferation (−/+)	G1-S phase entry, G2-M cell cycle	[2, 11]
FOXO1, FOXO3, and FOXO4	Apoptosis (+)	Extrinsic and intrinsic apoptotic pathways	[12–15]
FOXO1, FOXO3	Metabolism (+)	Glucose-6-phosphatase Phosphoenolpyruvate carboxykinase PGC1 Apolipoprotein C-III	[2]
FOXO3	Differentiation (+/−)	B cell translocation gene 1 DNA binding 1 Myostatin, neurogenin 3, and NK homeobox factor 6.1	[16–18]
FOXO1, FOXO3	Oxidative stress (+)	Glutathione, selenoprotein P, manganese superoxide dismutase, and peroxiredoxin III	[19–23]
FOXO1, FOXO3	Atrophy (+)	Gabarapl1, Atg12, calcineurin/nuclear factor, and atrogin-1	[24, 25]
FOXO1	Inflammation (+)	IL-1*β*, IL-6, IL-12, and Tlr4	[26–30, 30]
DAF-16, FOXOs	Aging (−)	P53, SIRT1, NF-*κ*B, MnSOD, heat-shock proteins, and antimicrobial agents	[31–33]
DAF-16, FOXO3	Reproduction (−)	Cell cycle inhibitor p27 enzyme galactose-1-phosphate uridyltransferase (Galt) Prostaglandins	[34, 35]

Listed are cellular functions and related transcriptional targets or pathways that have been reported to be directly regulated by FOXO transcription factors. The effect of FOXO is depicted by increasing (+) or decreasing (−) the indicated cellular activity.
